# Are carnivorous plants mixotrophic?

**DOI:** 10.1111/nph.70260

**Published:** 2025-06-03

**Authors:** Qianshi Lin, Shuhan Sarah Yin, Martín Mata‐Rosas, Enrique Ibarra‐Laclette, Tanya Renner

**Affiliations:** ^1^ Department of Entomology The Pennsylvania State University 501 ASI Building University Park PA 16802 USA; ^2^ Department of Biology Lakehead University 955 Oliver Rd Thunder Bay ON P7B 5E1 Canada; ^3^ Red Manejo Biotecnológico de Recursos, Instituto de Ecología, A.C. Carretera Antigua a Coatepec No. 351, Col. El Haya, C.P. Xalapa Veracruz 91073 Mexico; ^4^ Red de Estudios Moleculares Avanzados, Instituto de Ecología, A.C. Carretera Antigua a Coatepec No. 351, Col. El Haya, C.P. Xalapa Veracruz 91073 Mexico

**Keywords:** adaptation, carbon (C) assimilation, heterotroph, mixotroph, nitrogen (N) assimilation, plant carnivory

## Disclaimer

The New Phytologist Foundation remains neutral with regard to jurisdictional claims in maps and in any institutional affiliations.

Carnivorous plants, which capture animal prey to thrive in nutrient‐poor environments, have independently originated 12 times in angiosperms (Fleischmann *et al*., 2018; Lin *et al*., 2021). These plants have evolved various types of traps, with sticky traps being the most common strategy (Freund *et al*., 2022). The sticky trap has independently evolved in at least six lineages, including the Caryophyllales and Lamiales (Freund *et al*., 2022), and all other trap types may have evolved from it (Albert *et al*., 1992; Fleischmann *et al*., 2018; Freund *et al*., 2022).

Most carnivorous plants with sticky traps use modified leaves to capture insects and photosynthesize simultaneously, which may diminish their photosynthetic performance (Wicke *et al*., 2014; Ross *et al*., 2016; Fu *et al*., 2023). Additionally, previous studies demonstrate that terrestrial carnivorous plants have a lower photosynthetic rate than noncarnivorous plants (Méndez & Karlsson, 1999; Ellison, 2006). This is consistent with plastid gene loss, a trait observed in all tested carnivorous lineages, except for *Brocchinia* (Gruzdev *et al*., 2019; Nevill *et al*., 2019; Fu *et al*., 2023). Most plastid gene loss in carnivorous plants occurs within the NDH (=NAD(P)H dehydrogenase‐like) complex, which is likely involved in photooxidative stress responses (Peltier *et al*., 2016; Yamori & Shikanai, 2016; Strand *et al*., 2019). Thus, it has been hypothesized that carnivorous plants assimilate organic carbon from prey to overcome environmental stress, which makes the NDH complex dispensable (Wicke *et al*., 2014; Silva *et al*., 2016, 2018; Fu *et al*., 2023). The degradation of photosynthetic functions and plastomes is also prevalent in heterotrophic plant lineages (Wicke *et al*., 2016; Graham *et al*., 2017), which include parasitic plants that obtain nutrients from plant hosts and mycoheterotrophic plants that rely on underground fungi (Merckx, 2012; Heide‐Jørgensen, 2008). Therefore, the degradation of photosynthetic functions and plastomes may suggest that certain species of carnivorous plants are mixotrophs that combine autotrophic photosynthesis with heterotrophic nutrition (Tĕšitel *et al*., 2018).

Carnivorous plants absorb essential nutrients like nitrogen (N) and phosphorus (P) from their animal prey, which suggests that they can also absorb organic carbon (C) like heterotrophic plants (Wicke *et al*., 2014; Ross *et al*., 2016; Fu *et al*., 2023). Former studies usually used a natural abundance of heavy isotopes to track the nutrient flow from animals to carnivorous plants (e.g. see Merckx, 2012; Klink *et al*., 2019), which may not be sensitive enough if the C transfer is low. By using a more sensitive labeled isotope method, direct uptake of carbon from prey has been demonstrated in several studies and fuels respiration in the Venus flytrap (Dixon *et al*., 1980; Greenway *et al*., 1990; Rischer *et al*., 2002; Kruse *et al*., 2014, 2017; Gao *et al*., 2015; Fasbender *et al*., 2017). However, further experiments comparing the ratio of C and N transfer from isotopically labeled prey are still needed to determine whether heterotrophy is widespread across multiple carnivorous plant lineages.

Here, we used double‐labeled (^13^C and ^15^N) insects to detect C transfer, which has been commonly used to confirm heterotrophy and trace the C flow in parasitic and mycoheterotrophic plants (e.g. see McKendrick *et al*., 2002; Merckx *et al*., 2010; Yang *et al*., 2012; Gebauer *et al*., 2016; Gomes *et al*., 2020). We also compared C transfer with N transfer across four carnivorous species belonging to two independent lineages. We used tissue‐cultured plants grown under the same conditions within the lab to diminish any factor of variability in natural conditions. We tested four carnivorous species with sticky traps, two *Drosera* (Caryophyllales) and two *Pinguicula* (Lamiales), to demonstrate if heterotrophy is common among carnivorous plants and if there is any differentiation of heterotrophy among lineages.

**Fig. 1:**
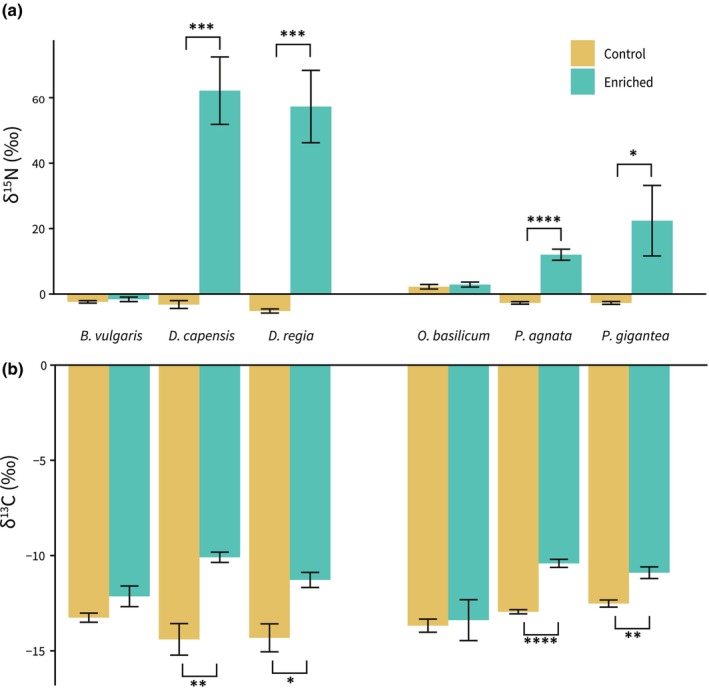
Average δ^15^N and δ^13^C in carnivorous plants compared to noncarnivorous relatives. Comparison of δ^15^N (a, mean ± SE) and δ^13^C (b, mean ± SE) among four carnivorous species (*Drosera capensis*, *Drosera regia*, *Pinguicula agnata* and *Pinguicula gigantea*) and two noncarnivorous relatives (*Beta vulgaris* and *Ocimum basilicum*), following exposure to ^15^N/^13^C‐labeled or unlabeled fruit flies for 1 wk. Significantly different values (*P* < 0.05) between labeled and unlabeled treatments are noted by an asterisk (*, *P* < 0.05; **, *P* < 0.005; ***, *P* < 0.0005; ****, *P* < 0.00005). (a) Average δ^15^N between control and enriched treatments in *D. capensis* and *D. regia* compared to *B. vulgaris*, and *P. agnata* and *P. gigantea* compared to *O. basilicum*. Carnivorous species show significant δ^15^N uptake between control and enriched treatments when compared to a noncarnivorous relative. (b) Average δ^13^C between control and enriched treatments in aforementioned species. Carnivorous species show significant δ^13^C uptake between control and enriched treatments when compared to a noncarnivorous relative.

**Fig. 2:**
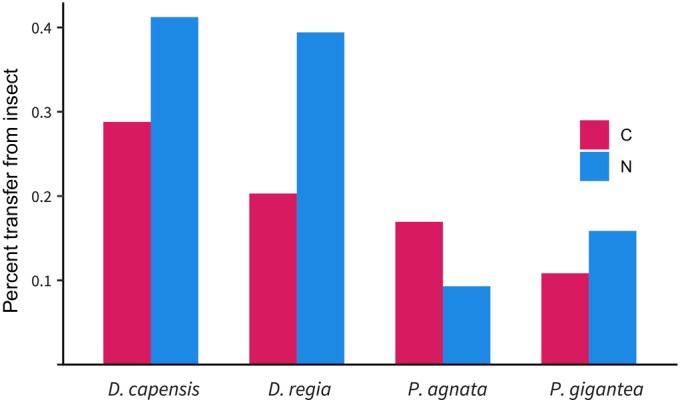
Percent carbon (C) and nitrogen (N) transfer from prey to carnivorous plants. Percentage of C and N transferred from fruit flies to nonfed traps in four carnivorous species (*Drosera capensis*, *Drosera regia*, *Pinguicula agnata* and *Pinguicula gigantea*) after 1 wk. Generally, percent transfer of C is roughly half that of N in all species except for *P. agnata*, where percent transfer of C is nearly twice that of N.

The δ^15^N for all control and carnivorous plants was within −5.19‰ to 2.22‰. One week after feeding the plants with labeled insects, the control plants showed no significant increase in δ^15^N levels; however, all four carnivorous species exhibited a large and significant increase in δ^15^N, with *Drosera capensis* experiencing an increase up to 62.16‰ (Fig. 1). The two *Drosera* species experienced a greater increase in δ^15^N levels than the two *Pinguicula* species. A simple mixing model suggests that up to 0.41% of N was transferred from insects to plants, and the two *Drosera* species got more than twice as much N as the two *Pinguicula* species (Fig. 2).

The δ^13^C for all control and carnivorous plants was within −14.40‰ to −12.52‰. One week after feeding the plants with labeled insects, the control plants showed no significant increase in δ^13^C levels, but all four carnivorous species exhibited a significant increase in δ^13^C, with *D. capensis* experiencing an increase up to −10.09‰. A simple mixing model suggests that up to 0.29% of C was transferred from insects to plants. However, δ^13^C uptake is generally less than that of δ^15^N, except in the case of *Pinguicula agnata*, which has a more significant average δ^13^C uptake than δ^15^N (Fig. 2).

Like animal stomachs, carnivorous plants use their traps to digest prey and enzymes similar to animal pepsin to break down proteins (Freund *et al*., 2022). The uptake of mineral nutrition like N and P has been well demonstrated in carnivorous plants (Adamec *et al*., 2018). It has also been shown that carnivorous plants take up organic substances like amino acids from prey or solutions (Juniper *et al*., 1989; Karagatzides *et al*., 2009). However, even though C uptake has been occasionally documented from absorption of amino acids (Dixon *et al*., 1980; Rischer *et al*., 2002), the magnitude of C uptake has yet to be quantified across different lineages; thus, whether carnivorous plants are heterotrophic is still undetermined (Givnish *et al*., 2018).

Our results clearly show that four carnivorous species across two independent lineages assimilate C from prey, which strongly suggests that heterotrophy may be common in carnivorous plants (Fig. 1). It has been shown that *Dionaea* uses prey‐derived amino acid C to fuel respiration and can be considered heterotrophic (Fasbender *et al*., 2017), but C is localized to the fed trap and petiole and does not move systemically through the plant (Kruse *et al*., 2017). However, here we clearly show that C is transferred from prey to nonfed traps in *Drosera* and *Pinguicula*. The utilization of C assimilation may vary among different carnivorous lineages, and the extent of which should be investigated in the future.

Our results also show that the uptake of C is around half the amount of N in all carnivorous species tested, except for *P. agnata* (Fig. 2). Although carnivorous plants obtain more N than C from prey, C uptake is not trivial. In *P. agnata*, the uptake of C is even higher, about twice that of N, which indicates the magnitude of carbon assimilation, or the level of heterotrophy, may vary among carnivorous plants. Interestingly, alpha‐amylase has been identified in the digestive fluid of *P*. × *Tina* (a hybrid of *P. agnata* and *P. zecheri*) (Kocáb *et al*., 2020). This enzyme has not been found to be present in the digestive fluid proteomes of other carnivorous genera and may point to heterotrophic tendencies within *Pinguicula*. It has been shown that aquatic *Utricularia* can grow in complete darkness with a carbohydrate‐rich medium (Harder, 1970) and prey‐derived organic carbon is important for aquatic carnivores when carbon dioxide (CO_2_) and light are limited (Adamec, 1997). This indicates that some carnivorous plants may acquire more C from prey than others or that they can become more heterotrophic under certain conditions, which also varies among hemiparasitic plants (Těšitel *et al*., 2010). In tuberous *Drosera*, for example, variants lacking stalked tentacles were found to be incapable of C uptake from prey, while normal variants with stalked tentacles absorbed and translocated C throughout the plant body and daughter rhizome (Dixon *et al*., 1980). In the case of *Pinguicula*, certain species are heterophyllous and form either a noncarnivorous winter rosette or hibernaculum (Legendre, 2000; Shimai *et al*., 2021), which may result in seasonal differences in C uptake. Furthermore, the production of carnivorous organs can be a phenotypically plastic trait influenced by nutrient availability (Dixon *et al*., 1980; Ellison & Gotelli, 2002; Englund & Harms, 2003; Thorén *et al*., 2003; Bott *et al*., 2008; Pavlovič *et al*., 2010; Gao *et al*., 2015). Research into the impact of environmental conditions on heterotrophy, and subsequently, on the evolution of related gene families in the carnivorous plants needs to be tested. Further insight could be taken from hemiparasitic *Pedicularis* (Orobanchaceae), a genus of relatively recent heterotrophs in which variation in plastome degradation exists (Li *et al*., 2021).

It is unsurprising that carnivorous plants assimilate organic C from prey and have some level of heterotrophy, considering they can easily obtain nutrients following digestion. However, since all carnivorous plants investigated can photosynthesize (Pavlovič, 2022), they should be treated as partially heterotrophic or mixotrophic, similar to hemiparasitic and partially mycoheterotrophic plants. Therefore, we might expect that the magnitude of C uptake may differ across carnivorous lineages. We suggest repeating similar experiments to investigate levels of heterotrophy in additional species outside the Caryophyllales and Lamiales. Although we confirmed that C was transported from fed to unfed traps, further research is warranted to determine how carnivorous plants use prey‐derived C and what proportion of total C comes from photosynthesis.

## Materials and Methods

### Plant material

We selected species circumscribed to two distantly related lineages of carnivorous plants, Caryophyllales: Droseraceae (*Drosera capensis* L. and *Drosera regia* Stephens) and Lamiales: Lentibulariaceae (*Pinguicula agnata* Casper and *Pinguicula gigantea* Luhrs). Carnivorous plants were sourced from The Institute of Ecology (Instituto de Ecología, A.C.). Plants were propagated by tissue culture on ½‐strength Murashige & Skoog (½MS) media (50 ml per jar) with added activated charcoal (media recipe: ½MS, 20 g l^−1^ sucrose, 8 g l^−1^ agar, 1 g l^−1^ activated charcoal, 3 ml l^−1^ PPM, pH 5.5) to reduce genetic variability among individual plantlets. We chose *Beta vulgaris* L. (Caryophyllales; US National Plant Germplasm System; accession no PI531253) and *Ocimum basilicum* L. (Lamiales; US National Plant Germplasm System; accession no PI652061) as noncarnivorous plant controls. Control plants were grown from seeds on the same MS tissue culture media as carnivorous plants.

### Isotope experiment

We fed 200 fruit flies (*Drosophila melanogaster* Meigen) with ^13^C and ^15^N‐labeled amino acids (100 mg algal amino acid mixture, Sigma‐Aldrich, added to 100 g standard *D. melanogaster* medium). Another 100 fruit flies were fed a normal standard *D. melanogaster* medium as a control. Fruit flies were frozen before experimentation. We placed three labeled fruit flies on the most recent fully developed leaf of each plant, 10 plants for each carnivorous species, and five for each noncarnivorous species. A 0.05% Tween‐20 solution was used to fix insects lightly to the leaf. Three unlabeled fruit flies were also placed on five plants for each species as controls. We kept plants with insects in the jar and collected whole leaves untouched by insects after 1 wk.

For both *Pinguicula* species and *D. regia*, we collected three to five leaves per plant that were untouched by the insects and found lower in the whorl (older leaves). For *D. capensis*, we collected all leaf material that had not come in direct contact with the labeled insect.

### Data collection and analysis

All samples were dried by silica gel, ground to powder, and sent to the University of California Davis Stable Isotope Facility (SIF), except for the unlabeled carnivorous plant and unlabeled *D. melanogaster* samples, which were sent to the Laboratory for Isotopes and Metals in the Environment (LIME) at The Pennsylvania State University for isotopic analysis. δ^13^N and δ^15^N were analyzed using a PDZ Europa ANCA‐GSL elemental analyzer interfaced to a PDZ Europa 20‐20 isotope ratio mass spectrometer (Sercon Ltd, Cheshire, UK) at the SIF, and a Thermo Delta V Isotope Ratio Mass Spectrometer with Elemental Analyzer (Costech 4010, Costech Analytical Technologies Inc., Valencia, CA, USA) coupled to a Conflo IV interface at the LIME. δ^13^C was expressed in units of per mil (‰) where δ = [(*R* sample/*R* standard) − 1] × 1000, *R* = ^13^C : ^12^C, with carbon in international standards Vienna Pee Dee Belemnite as the standard. δ^15^N was expressed in units of per mil (‰) where δ = [(*R* sample/*R* standard) − 1] × 1000, *R* = ^15^N : ^14^N, with nitrogen in air as the standard. The precision was typically ±0.2‰ for δ^13^C and ±0.3‰ for δ^14^N (Supporting Information Dataset S1).

We used a mixing model (Dataset S1) to calculate the fraction of the C/N content of plants or tissues derived from insect C/N:

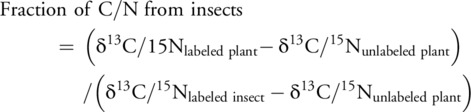




### Statistical analysis

In order to run a *t*‐test, data were determined to be normal and the variances unequal (see isotope.ttest.R, Dataset S2). Then we utilized Welch's two sample *t*‐test to determine statistical significance between average δ^13^C and δ^15^N measured in control and enriched treatments, for each species (Datasets S1, S2). Bar charts were created in R v.4.3.2 (R Core Team, 2023) using ggplot2 (Wickham, 2016) and figures were finalized in Adobe Illustrator (isotope.barcharts.R, Dataset S2).

## Competing interests

None declared.

## Author contributions

QL conceived of the project. QL and SSY conducted experiments, collected data, and analyzed results. SSY prepared the figures. QL, SSY and TR interpreted results and wrote the initial draft of the manuscript. QL, SSY, MM‐R, EI‐L and TR made comments on and final edits to the manuscript. TR contributed funding for the project. QL and SSY share joint first authorship of this work.

## Supporting information


**Dataset S1** Raw data for δ^13^C and δ^15^N (sheet 1), results of simple mixing model (sheet 2), and statistical results for Welch's *t*‐test (sheet 3).

**Dataset S2** R scripts for Welch's *t*‐test assumptions, statistical analysis, and bar chart generation. R software (R Core Team, 2023) is required to run scripts associated with Dataset S2. The software package ggplot2 (Wickham, 2016) is required to run the isotope.barcharts.R script.
Please note: Wiley is not responsible for the content or functionality of any Supporting Information supplied by the authors. Any queries (other than missing material) should be directed to the *New Phytologist* Central Office.

## Data Availability

Supporting Information is available for this paper.
